# Integrated slice-specific dynamic shimming for whole-body diffusion-weighted MR imaging at 1.5 T

**DOI:** 10.1007/s10334-020-00898-6

**Published:** 2020-12-23

**Authors:** Sarah McElroy, Jessica M. Winfield, Olwen Westerland, Geoff Charles-Edwards, Joanna Bell, Radhouene Neji, Alto Stemmer, Berthold Kiefer, Matthew Streetly, Vicky Goh

**Affiliations:** 1 Clinical Imaging and Medical Physics, Guy’s and St Thomas’ Hospital, London, UK; 2 MR Research Collaborations, Siemens Healthcare, Frimley, UK; 3 MR Application Predevelopment, Siemens Healthcare, Erlangen, Germany; 4 Clinical Haematology, Guy’s and St Thomas’ NHS Foundation Trust, London, UK; 5 Department of Cancer Imaging, School of Biomedical Engineering and Imaging Sciences, King’s College London, London, UK

**Keywords:** Whole-body imaging, Diffusion-weighted MRI, Oncology, Artefacts, Echo-planar imaging

## Abstract

**Objective:**

To compare integrated slice-specific dynamic shim (iShim) with distortion correction post-processing to conventional 3D volume shim for the reduction of artefacts and signal loss in 1.5 T whole-body diffusion-weighted imaging (WB-DWI).

**Methods:**

Ten volunteers underwent WB-DWI using conventional 3D volume shim and iShim. Forty-eight consecutive patients underwent WB-DWI with either volume shim (*n* = 24) or iShim (*n* = 24) only. For all subjects, displacement of the spinal cord at imaging station interfaces was measured on composed *b* = 900 s/mm^2^ images. The signal intensity ratios, computed as the average signal intensity in a region of high susceptibility gradient (sternum) divided by the average signal intensity in a region of low susceptibility gradient (vertebral body), were compared in volunteers. For patients, image quality was graded from 1 to 5 (1 = Poor, 5 = Excellent). Signal intensity discontinuity scores were recorded from 1 to 4 (1 = 2 + steps, 4 = 0 steps). A *p* value of < 0.05 was considered significant.

**Results:**

Spinal cord displacement artefacts were lower with iShim (*p* < 0.05) at the thoracic junction in volunteers and at the cervical and thoracic junctions in patients (*p* < 0.05). The sternum/vertebra signal intensity ratio in healthy volunteers was higher with iShim compared with the volume shim sequence (*p* < 0.05). There were no significant differences between the volume shim and iShim patient groups in terms of image quality and signal intensity discontinuity scores.

**Conclusion:**

iShim reduced the degree of spinal cord displacement artefact between imaging stations and susceptibility-gradient-induced signal loss.

**Supplementary Information:**

The online version contains supplementary material available at 10.1007/s10334-020-00898-6.

## Introduction

Whole-body MRI is accepted as the gold-standard imaging modality for suspected and newly diagnosed myeloma and plasmacytoma, and is recommended by the International Myeloma Working Group and the National Institute for Health and Care Excellence [1, 2]. Whole-body diffusion-weighted imaging (WB-DWI) was first introduced by Takahara et al. [3], and many studies have since shown the added benefit of this technique for diagnosis, staging and treatment response in oncology [4–6]. WB-DWI studies are generally acquired with a single-shot echo-planar imaging (EPI) sequence which is very efficient and motion-robust, combined with fat suppression to minimise artefacts. EPI is, however, prone to geometric distortion and signal loss, which are the result of phase accumulation in regions of high susceptibility gradient. This is especially pronounced in regions of high susceptibility gradient, notably within the head (e.g. base of skull, sinuses), neck, and thorax (e.g. chest wall) [7–9]. Another drawback of conventional WB-DWI techniques is the spinal cord displacement artefact which is often apparent when WB-DWI is viewed as a sagittally reformatted image (as is common in clinical practice) [10]. The different shim settings which are applied for the acquisition of each station result in an apparent spatial displacement at the junction of adjacent stations when reformatted in the sagittal plane.

The presence of distortion, signal loss, and artefacts in WB-DWI studies can hamper evaluation of bone disease and cord compression in myeloma [7, 10, 11]. Several techniques have been developed to overcome these issues. The spinal cord displacement artefact can be minimised by applying the shim settings of one station to all subsequent stations [12]. However, this can lead to other image quality issues, such as more pronounced chemical shift artefacts and failure of frequency-selective fat suppression [10]. Distortion and/or signal loss can be improved by reducing the phase field of view [13–16], reducing the echo spacing [17], using the reverse gradient polarity method [18], or by post-processing [19]. Each of these techniques has been shown to improve EPI image quality, but residual distortion,signal loss and artefacts remain due to B0 inhomogeneity. Slice-specific dynamic shimming is a technique which could improve the limitations of EPI by reducing B0 inhomogeneity [20]. At each station, the prototype integrated slice-specific dynamic shimming method (iShim) used in this work [21] acquires a 2-dimensional gradient-recalled echo sequence before the WB-DWI acquisition, which is used to calculate a field map for each slice. From the field maps, a centre frequency and linear shim terms are determined for each slice. The centre frequency and linear shim terms are then set (dynamically updated) before the acquisition of each EPI slice. This shimming technique is restricted to first-order shimming, with higher order shims set to a constant, patient-independent value, as shim current settling times for higher order shimming would exceed the minimum time between slice acquisitions. In particular, the values of the higher order shim currents are not modified between the acquisition of the field map and the actual scan. The acquired field map, corrected for first-order shim settings, can, however, be used to correct for residual distortion [19, 21]. This combination of slice-specific shimming and post-processing has an advantage over post-processing alone, which cannot compensate for signal loss [21].

A number of studies have reported improvement in signal-to-noise ratio and reduction in geometric distortion at 3 T for prostate [22], head and neck [8, 9], thyroid [23], breast [24], and bladder [25] for slice-specific dynamic shimming over conventional volume-based shimming. Slice-specific dynamic shimming has also been shown to be effective in reducing distortion, signal loss, and spatial displacement artefacts for WB-DWI at 3 T [26]. There are, however, few studies investigating the impact of slice-specific shimming at 1.5 T, which represents a significant proportion of clinical scanners installed [27]. While DWI is more prone to distortion, signal loss, and artefacts at 3 T, these drawbacks are also present at 1.5 T [12, 19, 20]. Therefore, the aim of our study was to investigate the impact of slice-specific shimming with distortion correction post-processing for the reduction of artefacts and signal loss compared to conventional 3D volume shimming for 1.5 T WB-DWI.

## Methods

### Subjects

Ten healthy volunteers (three male, seven female, median age 34 years, age range 24–38 years) were recruited for the study, following approval by the NRES London Bridge research ethics committee (study number 01/11/12) and providing written informed consent. Additionally, haematology patients undergoing WB-MRI for suspected myeloma were retrospectively identified from our institutional database (scan dates between January 2017 and January 2018). Twenty-four consecutive patients scanned with the conventional 3D volume shim WB-DWI sequence (13 male, 11 female, median age 72 years, age range 49–87) and 24 consecutive patients scanned with the iShim WB-DWI sequence (11 male, 13 female, median age 62 years, age range 28–87) were included. A review board waiver was granted for the analysis of anonymised patient data.

### Magnetic resonance imaging

All subjects were scanned on a 1.5 T MR scanner (MAGNETOM Aera, Siemens Healthcare, Erlangen, Germany, VE11C software). Volunteers and patients were positioned in a head-first, supine position and scanned with a standard protocol consisting of seven axial imaging stations (each consisting of 40 × 5 mm contiguous slices), covering from skull vertex to mid-calf. At each imaging station, T1-weighted 3D Dixon FLASH (VIBE), T2-weighted 2D single-shot turbo spin echo (HASTE), and 2D single-shot echo-planar DWI sequences with conventional 3D volume shim and/or with prototype iShim functionality [21] were acquired. All DWI acquisitions were acquired in free-breathing. The MR table was automatically positioned at the magnet isocenter for each station. Apart from the shimming method (either iShim or 3D volume shim, or both, depending on cohort), the DWI acquisition parameters were identical (Table 1). Patients also underwent sagittal 2D T1-weighted and STIR TSE imaging of the spine, as well as coronal T1-weighted pre- and post-contrast VIBE imaging.

**Table 1:** Acquisition parameters for in-vivo studies

Acquisition plane	Axial
TR/TE (ms)	6360/67
FOV (mm^2^)	500 × 500
Acquisition matrix	128 × 128
Slice thickness (mm)	5
Slice gap (mm)	0
Number of slices per station	40
Number of stations	7
In-plane acceleration	GRAPPA 2, integrated reference lines: 42
Fat suppression	STIR (TI = 180 ms)
Receiver bandwidth (Hz/pixel)	2300
Diffusion mode^a^	3-scan Trace
B values (s/mm^2^)	50, 900
NSA	2 for *b* = 50 s/mm^2^ 5 for *b* = 900 s/mm^2^
Diffusion gradient scheme	Monopolar (single refocused spin echo diffusion sensitizing)
Dynamic field correction	On
Composing algorithm	Diffusion
Receiver coils	Head/neck matrix 2 × body matrix Spine matrix Peripheral angio matrix
Total acquisition time^b^	19:30 (volume shim) 23:30 (iShim)

*TR* repetition time, *TE* echo time, *FOV* field of view, *STIR* short-tau inversion recovery, *TI* inversion time, *NSA* number of signal averages

^a^3-scan trace acquires three orthogonal oblique gradient directions sequentially

^b^Small increase in imaging time for iShim as a result of additional shimming and frequency adjustment

### Image analysis

Absolute spinal cord displacement artefact was measured in all subjects at each junction between imaging stations 1 and 4 (cervical, thoracic and lumbar junctions), all of which include the spinal cord in most individuals. In each case, the distance was measured from the anterior aspect of the spinal cord on the lower station to the anterior aspect of the spinal cord on the upper station.

A sternum-to-vertebra signal intensity ratio (SIR) was calculated to assess signal loss in a region of high susceptibility gradient. Circular regions of interest (ROIs) of 1 cm^2^ were drawn over the sternum and a region of relatively low susceptibility gradient (vertebral body), both at the same cranio-caudal position, on a mid-sagittal slice of all volunteer scans. The average signal intensity in these ROIs was recorded and the sternum-to-vertebra SIR was calculated as a ratio of these measurements. A higher SIR corresponds to lower signal loss in the sternum, which may be achieved with improved shimming in this region of high susceptibility gradient. These measurements were performed on volunteer images only since the presence of pathology could bias the results of these measurements in patient studies.

Image quality was graded qualitatively in patients from 1 to 5 (1 = poor, 2 = unsatisfactory,3 = satisfactory, 4 = very good, 5 = excellent), and signal intensity discontinuity scores, describing signal intensity discontinuity between adjacent bed positions, were recorded as 1–4, (1 = 2 + steps, 2 = 2 steps, 3 = 1 step, 4 = no steps) [26].

Spinal cord displacement measurements were carried out three times in total in healthy volunteers: twice by an MR physicist and once by a radiologist, to enable characterisation of the intra- and inter-observer reproducibility of this method.

Signal intensity ratios in volunteers were measured by an MR physicist. Two radiologists with 10 years and 5 years of whole-body MRI experience independently analysed the patient studies. All analysis was performed on sagittal inverted greyscale images reconstructed from axial *b* = 900 s/mm^2^ images (reflecting standard reporting practice at our institution), using the MR scanner manufacturer's post-processing software (syngo.via VB20A; Siemens Healthcare, Erlangen, Germany).

### Statistical analysis

All statistical analysis was carried out using Microsoft Excel 2018. As absolute spinal cord displacement measurements are not normally distributed, the Wilcoxon signed-rank test was used to test whether the spinal cord displacement measured on the iShim DWI sequence was different from the displacement measured on the volume shim DWI sequence in volunteers. Bland–Altman analysis was used to determine the intra- and inter-observer reproducibility of spinal cord displacement measurements. The Shapiro–Wilk test was used to test for normality of SIR measurements. A two-tailed paired *t* test was used to compare the difference in SIRs between the iShim and volume shim DWI sequences in volunteers. The Mann–Whitney *U* test was used to test the difference in spinal cord displacement, image quality scores, and signal discontinuity scores between iShim and volume 3D shim DWI sequences in patients. The value *p* < 0.05 was considered statistically significant in all statistical tests.

## Results

**Fig. 1:**
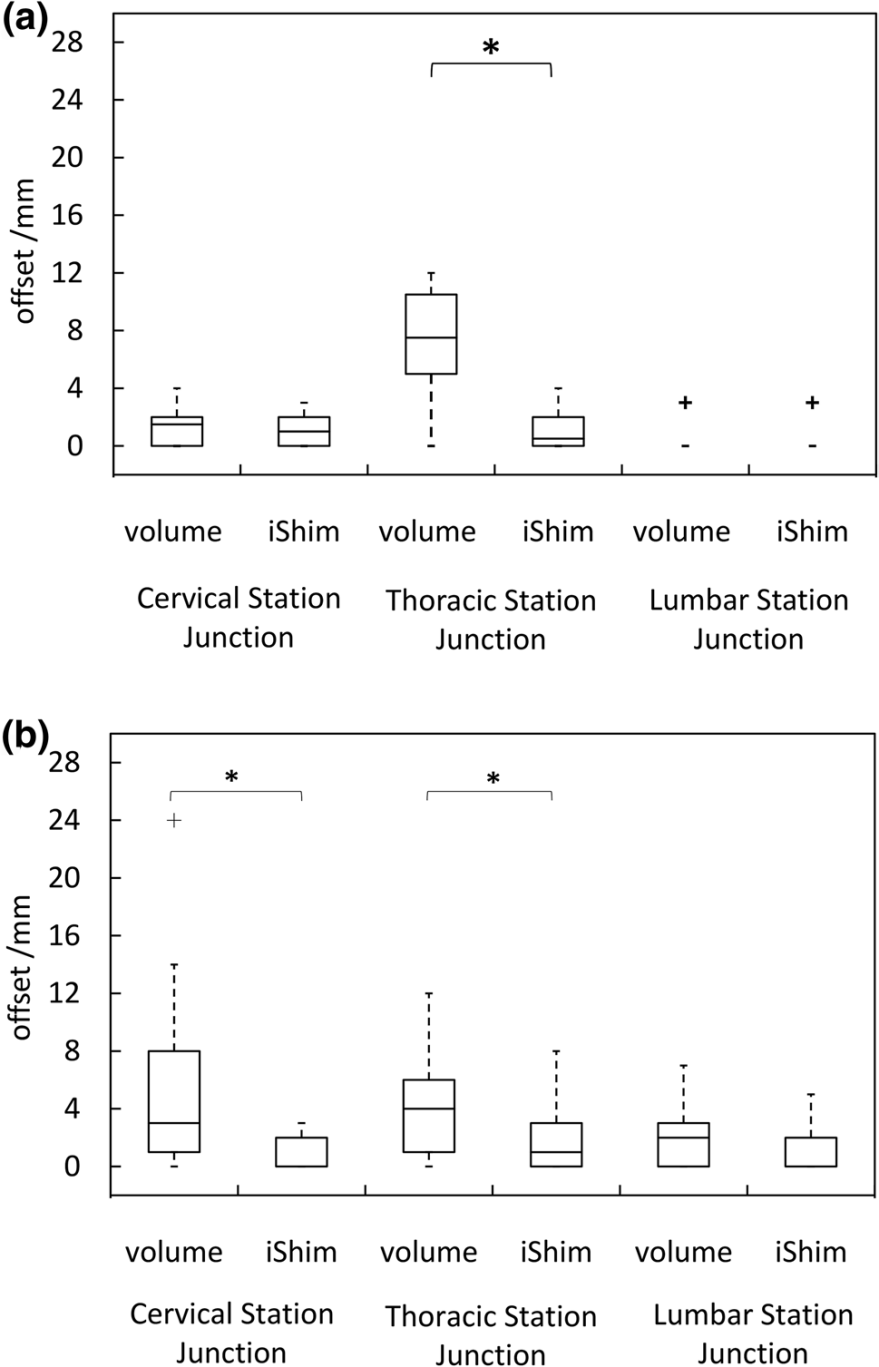
Spinal cord displacement offsets measured on sagittally reformatted images composed from axially acquired *b* = 900 s/mm^2^ images. **a** Boxplots showing offset in positions of spinal cord at the cervical and thoracic station junctions in ten healthy volunteers. Displacement at the lumbar station junction was measured in seven volunteers as the spinal cord did not extend to the lumbar station junction in three volunteers. The values for the median and quartiles 1 and 3 for this station were equal to zero for this station, resulting in no ‘box’ for this plot. **b** Boxplots showing offset in positions of spinal cord at cervical, thoracic and lumbar station junctions in 24 patients scanned with the conventional 3D volume shim WB-DWI sequence and 24 patients scanned with the iShim WB-DWI sequence. Offsets were measured on sagittally reformatted images composed from axially acquired *b* = 900 s/mm^2^ images

**Fig. 2:**
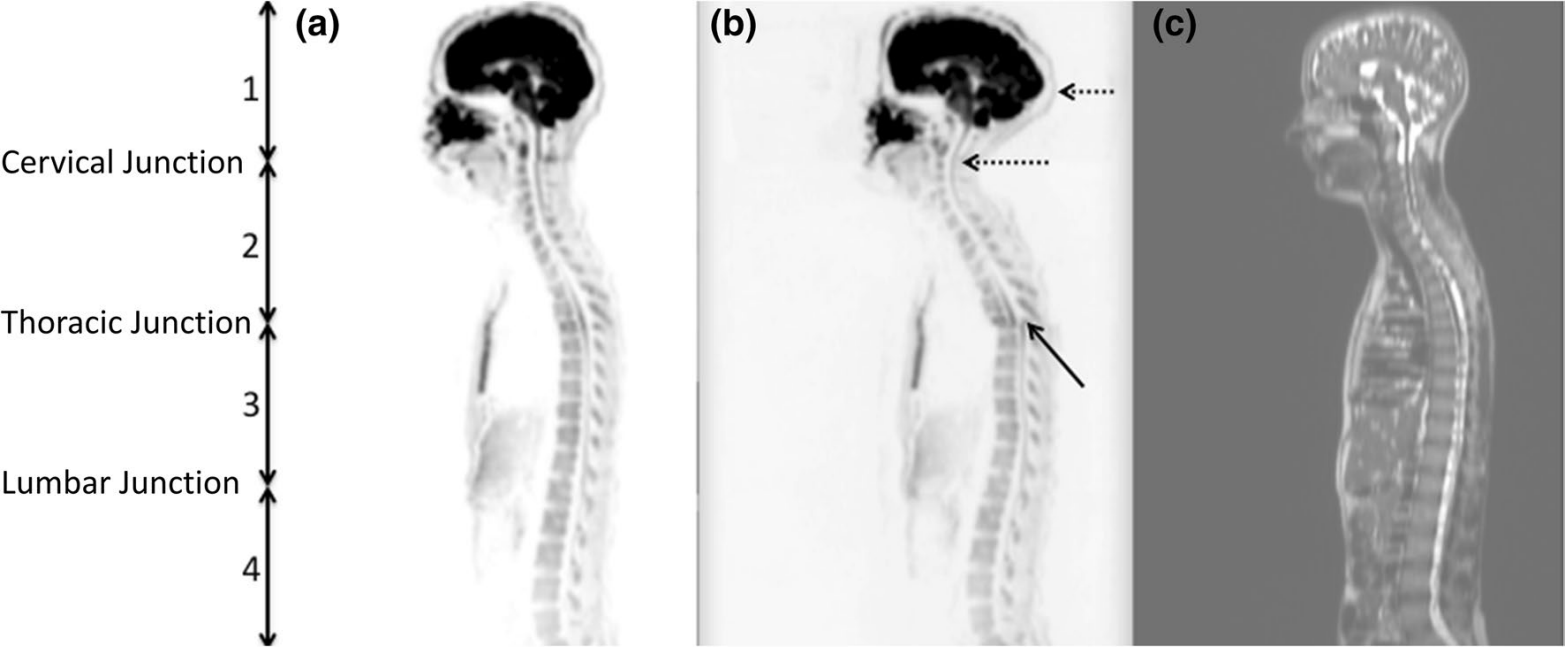
Sagittally reformatted composed images of volunteer study. **a** *b* = 900 s/mm^2^ images acquired using iShim. **b** *b* = 900 s/mm^2^ images acquired using volume shim. **c** T2-weighted HASTE image as an anatomical reference image with no significant distortion. Locations of stations (1–4) are shown by arrows on left-hand side. Solid arrow shows offset in positions of spinal cord at the thoracic station junction in diffusion-weighted images acquired using volume shim. Dashed arrows show distortion of spinal cord and head in upper station

**Fig. 3:**
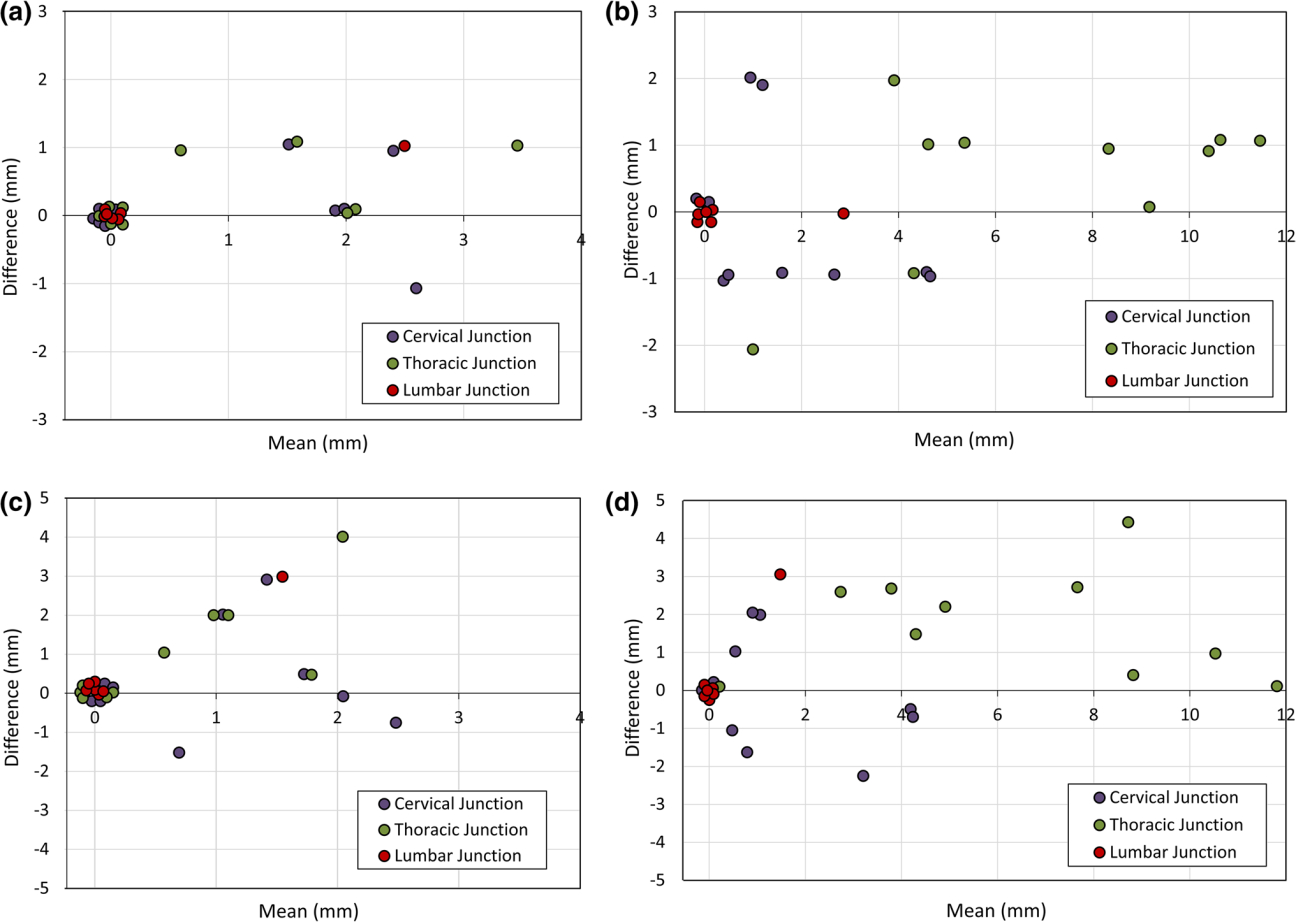
Bland–Alman plots showing intra-observer reproducibility (**a**, **b**) and inter-observer reproducibility (**c**, **d**) of spinal cord displacement artefact measurements in healthy volunteers,using the iShim shim DWI sequence (**a**, **c**) and the volume shim sequence (**b**, **d**). Overlapping points have been marginally offset for clarity

**Fig. 4:**
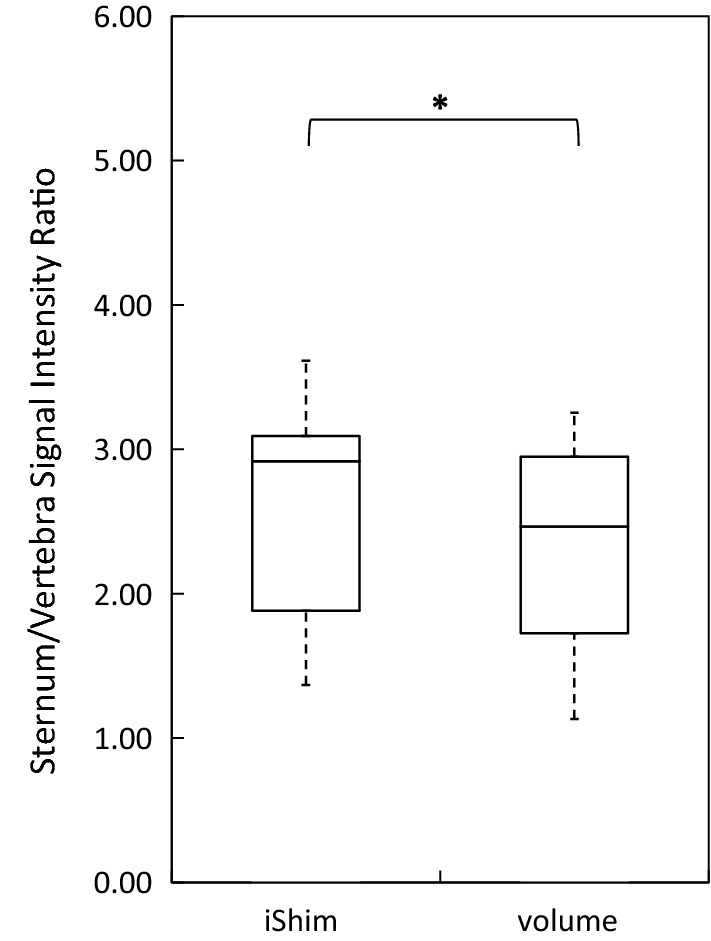
Boxplots showing sternum-to-vertebra signal intensity ratio in ten healthy volunteers. Signal intensity was measured on 1 cm^2^ circular ROIs drawn on sagittally reformatted images composed from axially-acquired *b* = 900 s/mm^2^ images

**Fig. 5:**
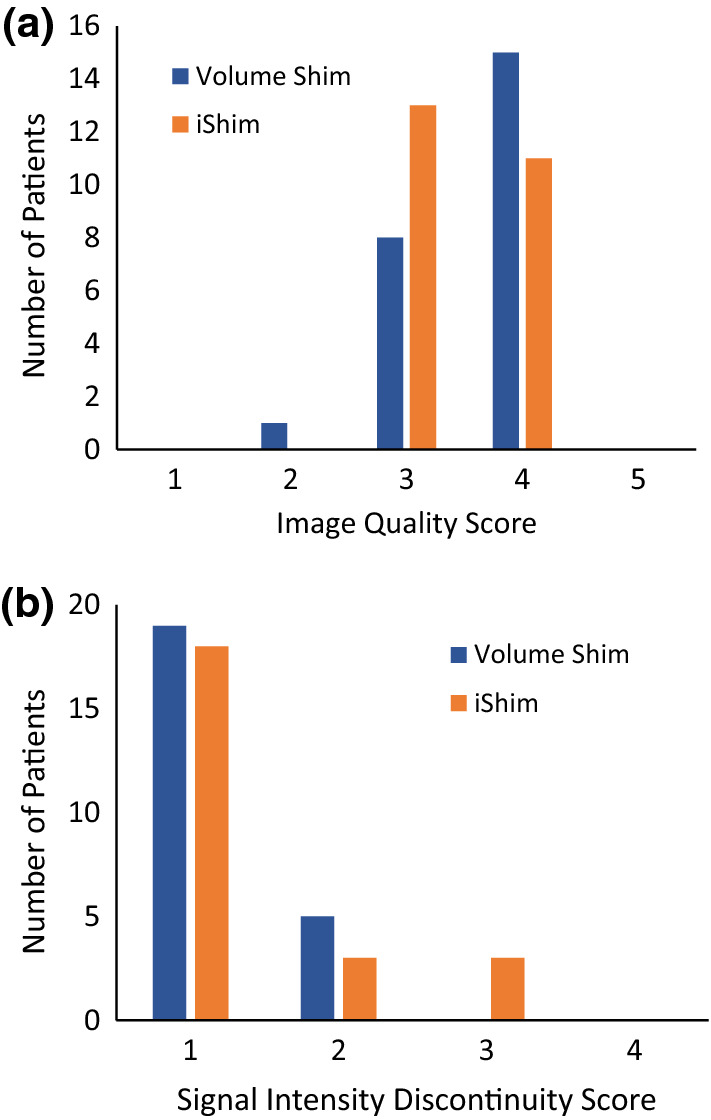
Qualitative assessment of images. **a** Image quality scores for patients. 1 = poor, 2 = unsatisfactory, 3 = satisfactory, 4 = very good, 5 = excellent. **b** Signal intensity discontinuity scores for patients. 1 = 2 + steps, 2 = 2 steps, 3 = 1 step, 4 = 0 steps

**Fig. 6:**
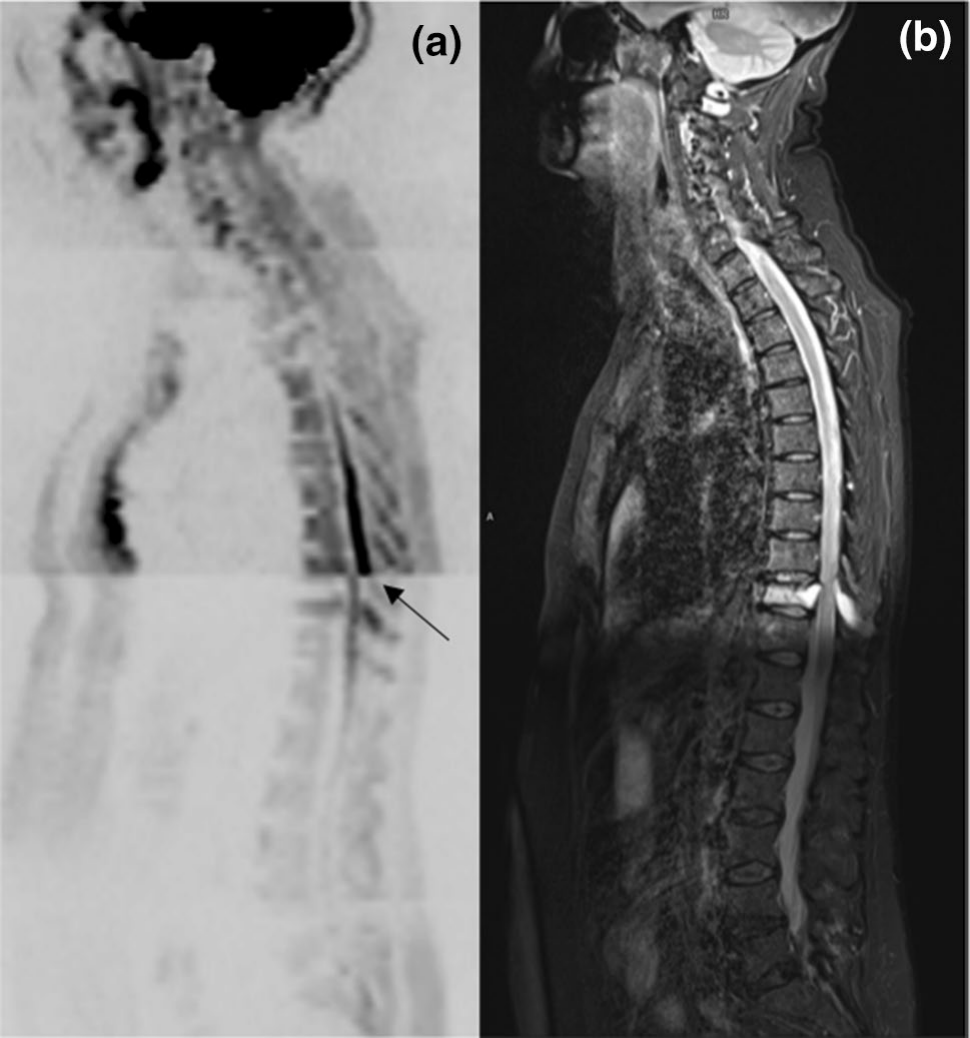
Sagittally reformatted composed images of patient study. **a** *b* = 900 s/mm^2^ images acquired using volume shim. **b** T2-weighted HASTE. Arrow indicates the spinal cord displacement artefact at the region of malignant cord compression

The distribution of spinal cord displacement offsets in the healthy volunteers and patients are presented in Fig. 1. The cord did not extend to the bottom station junction in three volunteers, so the results are shown for seven volunteers for this region. The iShim sequence demonstrated a significant reduction in spinal cord displacement compared with volume shim at the thoracic junction in volunteers (*p* < 0.05) and at the cervical junction (*p* < 0.05) and thoracic junction (*p* < 0.05) in patients. The maximum spinal cord displacement artefact was reduced from 12 to 4 mm using volume shim and iShim, respectively, in volunteers and from 24 to 8 mm using volume shim and iShim respectively in patients. Figure 2 shows an exemplary case demonstrating the reduction of spinal cord displacement at the thoracic junction on images of a volunteer. Markedly reduced distortion in the head and cervical spine stations was also noted on the iShim image. Bland–Altman plots of the intra- and inter-observer reproducibility of the spinal cord displacement artefact measurement in volunteers are shown in Fig. 3. The Bland–Altman mean difference and limits of agreement for intra- and inter-observer reproducibility are shown in Tables 2 and 3, respectively. The sternum-to-vertebra SIR was significantly increased in the iShim images compared with matched volume shim images (Fig. 4, *p* < 0.05, paired *t* test). The distribution of image quality and signal intensity discontinuity scores are shown in Fig. 5. There were no significant differences in image quality or signal intensity discontinuity scores between iShim and volume shim patient data sets (*p* > 0.05, Mann–Whitney *U* test). Figure 6a shows an example of spinal cord displacement artefact at the region of malignant thoracic cord compression in a patient with myeloma.

**Table 2:** Bland–Altman mean difference and limits of agreement in millimetres for the intra-observer reproducibility of spinal cord displacement artefact measurements in volunteers

	iShim	Volume shim
Cervical station junction	0.1 ± 1.1	0. ± 2.6
Thoracic station junction	− 0.3 ± 1.0	0.5 ± 2.4
Lumbar station junction	0.14 ± 0.75	0.0 ± 0.0

**Table 3:** Bland–Altman mean difference and limits of agreement in millimetres for the inter-observer reproducibility of spinal cord displacement artefact measurements in volunteers

	iShim	Volume shim
Cervical station junction	0.3 ± 2.5	− 0.1 ± 2.8
Thoracic station junction	0.9 ± 2.6	1.8 ± 2.8
Lumbar station junction	0.4 ± 2.2	0.4 ± 2.2

Limits of agreement are calculated as 1.96 × standard deviation

## Discussion

In this study, we compared iShim against volume shim for 1.5 T WB-DWI. Spinal cord displacement and susceptibility-related signal loss were lower using the iShim sequence compared to the volume shim sequence. Qualitative assessment of patient images showed no significant differences in image quality and signal intensity discontinuity scores. Spinal cord displacement artefact is caused by a discrepancy between the centre frequencies and shim terms of adjacent slices at the interface between two stations. Using the conventional 3D volume shimming technique, these values can vary quite significantly between adjacent slices at a station junction, since they are optimised for a whole station. With iShim, the centre frequency and shim terms are optimised for each slice, so the discrepancy between these values for any two adjacent slices is likely to be small.

The displacements measured in our study were lower than comparable measurements at 3 T. In one 3 T study [26], the maximum measured displacements, excluding outliers, were 20 mm and 16 mm using volume shim and iShim, respectively, in comparison to 14 mm and 8 mm in our study. This larger displacement measured at 3 T is likely caused by an increased B0 inhomogeneity at this field strength. Our study also employed retrospective field map based distortion correction (which was not used for the 3 T study), although a small study in ten patients did not demonstrate a significant reduction in spinal cord displacement artefact compared with slice-specific shimming alone (see Supplementary Material, Table 1). In clinical practice, it is desirable to reduce the presence of any significant artefact, the presence of which may result in reduced diagnostic confidence, increased reporting times, a need for repeat imaging, or, in the worst case, a misdiagnosis.

The 3 T WB-DWI iShim study also reached different conclusions compared with the current study with regards to image quality scores and signal intensity discontinuities. At 3 T, image quality scores showed an increase using iShim and signal intensity discontinuities decreased with iShim. This disparity between the two studies can be attributed to a lower degree of distortion and signal loss of DWI measurements at 1.5 T.

It was not possible to compare absolute signal intensity values in the sternum due to different post-processing steps resulting in different signal intensity scaling between iShim and volume shim images. Therefore, we used a relative assessment of signal loss in a region of high susceptibility gradient (sternum) to a region of lower susceptibility gradient (vertebra). A comparison of the signal intensity ratios measured with the two DWI sequences indicate a reduction of susceptibility-related signal loss in the sternum with the iShim sequence. Susceptibility-related signal losses could affect interpretation in cases of sternal disease. Reduced signal loss with iShim has also been demonstrated in other regions of the body with high susceptibility gradient, such as the neck [8].

In terms of workflow, dynamic shimming and retrospective distortion correction are integrated into the iShim sequence, maintaining a streamlined workflow, with a small increase in imaging time to allow for additional shimming and frequency adjustment. This has an advantage over other solutions, such as applying a fixed centre frequency across all stations, which require greater operator input.

Our study has some limitations. Due to patient comfort and scan-time restrictions, only a single DWI sequence was acquired in each patient. Therefore, the impact of the artefact on interpretation was limited as we could not directly compare the two sequences in each patient. Additionally, our study evaluated the performance of iShim as one vendor’s solution to reduce the distortion and signal loss associated with WB-DWI acquisitions. Therefore, the impact of dynamic shimming cannot be compared across different vendors.

## Conclusion

With the recommendation of WB-MRI as a first line investigation in a number of guidelines and an associated rise in the number of studies performed, image quality optimisation is essential, particularly as many of these patients may undergo serial studies, e.g. for response assessment. The results of our study demonstrate that the iShim technique can reduce spinal cord displacement artefacts and signal loss in WB-DWI scans at 1.5 T, which is currently the most commonly-used field strength for WB-MRI studies. There were no differences in terms of image quality scores and signal intensity discontinuities with the iShim technique compared to a conventional volume shim technique.

## Supplementary Information

Below is the link to the electronic supplementary material.

Supplementary file1 (PDF 110 KB)

## Abbreviations

DWI: Diffusion-weighted imaging
EPI: Echo-planar imaging
ROI: Region of interest
SIR: Signal intensity ratio
WB-DWI: Whole-body diffusion-weighted imaging
