# Supplementary material for: Integrated slice-specific dynamic shimming for whole-body diffusion-weighted MR imaging at 1.5 T
Source: MAGMA. Author manuscript; Available in PMC 2021 Aug 6. (PMC8338872; doi:10.1007/s10334-020-00898-6)
Supplement: Supplementary Information [file EMS119964-supplement-Supplementary_Information.pdf]

Integrated slice-specific dynamic shimming for whole-body diffusion-weighted MR imaging at 1.5T

Sarah McElroy[1], Jessica M Winfield[1], Olwen Westerland[1], Geoff Charles-Edwards[1], Joanna Bell[1], Radhouene Neji[2], Alto Stemmer[3], Berthold Kiefer[3], Matthew Streetly[4], Vicky Goh[1,5].

[1] Clinical Imaging and Medical Physics, Guy's and St Thomas' Hospital, London, UK.

[2] MR Research Collaborations, Siemens Healthcare, Frimley, UK.

[3] MR Application Predevelopment, Siemens Healthcare, Erlangen, Germany.

[4] Clinical Haematology, Guy's and St Thomas' NHS Foundation Trust, London, UK.

[5] Department of Cancer Imaging, School of Biomedical Engineering and Imaging Sciences, King's College London, UK.

Corresponding author:
Sarah McElroy
Clinical Imaging and Medical Physics, Guy's and St Thomas' Hospital, London, SE1 7EH,
Email: sarah.mcelroy@kcl.ac.uk

Supplementary Material: Investigation of the relative significance of prospective distortion correction using slice-specific shimming and retrospective field map based distortion correction on the reduction of spinal cord displacement artefact in whole body DWI at 1.5 T.

To assess the relative contributions of the slice-specific shimming and the retrospective field map based distortion correction on spinal cord displacement artefacts, a further 10 WB-DWI datasets were acquired with iShim in patients undergoing WB-MRI for suspected myeloma. The images were reconstructed both with and without retrospective field map based distortion correction. The spinal cord displacement artefact was measured at 3 station junctions as reported in the Methods section. The Wilcoxon signed rank test was used to test for a significant difference between the datasets. Results are presented in Table 1 as median (range) of spinal cord displacement artefact for each station.

Table 1: Results of spinal cord displacement artefact measurements in 10 patients, reported as median and (range) in millimetres.

	With Retrospective DC	Without Retrospective DC
Cervical Junction	0 (0-0)	0 (0-1)
Thoracic Junction	0 (0-1)	0.5 (0-3.5)
Lumbar Junction	0 (0-2)	1.5 (0-26)

While there was no significant difference found between the datasets with and without retrospective distortion correction, the maximum spinal cord displacement artefact was higher for the dataset without distortion correction. Therefore, a larger number of patients may lead to a more significant difference between these two datasets.
